# Two-Axial Measurement of the Angular Microdeflection of a Laser Beam Using One Single-Axis Sensor

**DOI:** 10.3390/s23229276

**Published:** 2023-11-20

**Authors:** Marek Dobosz, Michał Jankowski, Jakub Mruk

**Affiliations:** The Metrology and Biomedical Engineering Institute, Warsaw University of Technology, 02-525 Warsaw, Poland; marek.dobosz@pw.edu.pl (M.D.); mruk.kuba@gmail.com (J.M.)

**Keywords:** angular microdeflections, interferometric sensor, two-axial measurement, optical beam deflection sensing (OBDS), variable phase retarder

## Abstract

The majority of current methods for measuring the angular deflection of a laser beam enable measurement only in one selected plane. However, there are tasks in which measurements of laser beam deflections in 3D are required. In this paper, we present a way of enabling two-axial measurements of the deflection of a beam based on a single-axis sensor. The key idea is to direct a laser beam, alternately, into one of two arms of a measurement system. In the first arm, the beam is transmitted directly to the angular sensor, while in the second, the beam is directed to the sensor via a special optical element that rotates the plane of the beam deflection; in other words, this element changes the deflection in the horizontal plane into a deflection in the vertical plane, and vice versa. To alternate the path of the beam, a variable phase retarder and a polarising beamsplitter are used. The proposed technique was experimentally verified, and the results confirm its effectiveness.

## 1. Introduction

There are various methods of measuring the angular deflection of a laser beam, including approaches based on position (triangulation) [1,2,3], autocollimation [4,5,6,7], the critical-angle effect [8,9,10,11,12,13,14,15] and interference [16,17,18,19,20].

In triangulation-based methods the angular deflection of a laser beam is calculated from the linear displacement of the beam measured at a given distance. The sensitivity and accuracy of these methods are strongly related to dimensions of the setup or of the measurement device. For example, if the linear displacement of a beam is measured at a distance of 1 m, the linear resolution of 1 µm corresponds to the angular resolution of 1 µrad. Due to this drawback, applications of triangulation-based methods are limited. However, these methods enable two-axial measurement.

Autocollimation-based methods apply a similar principle, but an additional lens is added in front of the position-sensing detector. The distance between this lens and the detector is equal to the focal length of the lens, so an incident laser beam is focused on the position-sensing detector. These methods can be applied to achieve a very good resolution, even as small as single nanoradians, but at the cost of a very limited measurement range—several dozen µrad for the largest sensitivities [7]. This problem can be solved by the application of an optical frequency comb and a grating reflector, but such a solution works only in a single-axis measurement [5]. Moreover, autocollimators are usually relatively big and heavy, despite it being possible to achieve good results with focal lengths as small as 40 mm [7]. The autocollimators that are widely used in industry use non-coherent light and usually require a relatively big (e.g., from 30 to 50 mm in diameter) and heavy mirror. Smaller mirrors can be used, but the smaller the mirror, the less the emitted light that returns to the measurement device. Because of this, small mirrors are applied only in measurements at shorter distances. Autocollimators also enable two-axial measurement. Their accuracy is good—their maximum permissible errors are around 1 µrad or even smaller—and their measurement range can reach about 10 mrad or even more [21,22]. Unfortunately, high accuracy digital autocollimators are expensive.

In critical-angle-effect-based methods a laser beam is splitted and the intensity of transmitted light is compared with the intensity of reflected light. These methods have a good accuracy, but they can be applied only in cases of single-axis laser beam deflections. Any deflection in a perpendicular plane would disturb the measurement.

Interference-based methods are a broad group of various solutions which differ in metrological parameters. The sensor used in this research (described in Section 2.4) applies new interference-based methods described in [20] and has many advantages in comparison with competing techniques, and the presented research can be used with any single-axis angular deviation measurement system.

The methods mentioned above are methods of measurement of the angular deflection of a laser beam. These methods can be used to measure the angular displacement of a material object, for example, of a coordinate measuring machine ram [23]. In such applications, it is necessary to fix a mirror to the tested object, but in the case of the applied sensor this mirror can be very small and lightweight, only slightly bigger than the laser beam spot.

Described autocollimation-based or interference-based methods should not be confused with autocollimators and interferometers commonly used in industry. These devices are widely applied in measurement of the angular displacement of material objects, but their principles of operation are slightly different.

Most common industrial interferometers, like Renishaw XL-80 [24], do not measure the angular deflection of a laser beam. They measure the difference between linear displacements of two corner cube reflectors fixed to a tested object. Because the distance between these reflectors is known, it is possible to calculate the angular displacement of the tested object. In the case of coordinate measuring machines, multi-axis calibrators are also used. These devices enable measurement of several geometric errors of a machine at once, but there is a necessity to fix a heavy piece of equipment to the ram. This piece of equipment may have a mass of even more than half a kilogram [25]. Fixing such a mass to a tested object, for example, to the ram of a coordinate measuring machine, can seriously affect the measurement. In [23], it was shown that fixing an additional mass of 440 g to the ram changed the tested rotational error of a coordinate measuring machine by more than 5 µrad at a distance of 300 mm. The same problem may occur in the case of fixing a heavy mirror of an autocollimator to a tested object.

Some of these methods, such as the interference-based methods described in [20,23], enable only single-axis measurement, where the sensors are sensitive only to laser beam deflections in one selected plane. This type of deflection can be also presented as a rotation of the laser beam around a selected axis.

To describe how single-axis sensors respond to angular deflections of the laser beam in any chosen direction, the angular deflection of the beam needs to be expressed as a combination of two orthogonal components, i.e., angular deflections in two perpendicular planes, for example, vertical and horizontal. A single-axis sensor measures only one of these components: the deflection in the sensitivity plane of the sensor. The second component is not measured, and in some cases this decreases the accuracy of the sensor.

To solve this problem, a pair of single-axis beam deflection sensors can be used. In the most basic setup, the second sensor is rotated by 90° relative to the first sensor, so they are taking measurements in two perpendicular planes. This solution was applied in [23] to simultaneously measure the two rotational errors of a coordinate measuring machine. The idea presented in the present study involves the application of an optical element which rotates the plane of the beam deflection. When the beam passes through this element, its deflection in the horizontal plane is changed to a deflection in the vertical plane, whereas its deflection in the vertical plane is changed to a deflection in the horizontal plane. In this approach, both sensors may have the same orientation.

In the present paper, an optical setup for two-axial measurements of the angular deflections of a laser beam is presented. Only one single-axis beam deflection sensor is used, and the beam is alternately directed to the two arms of the optical setup. The first arm of the optical setup contains only a non-polarising beamsplitter and a polariser; the direction of the beam deflection in this arm remains the same as on entry to the setup. The second arm of the setup includes an optical element that rotates the beam deflection. The beams from both arms are passed to the same single-axis sensor. By alternating the path of the beam, alternating measurements of the beam deflections can be made in two perpendicular planes. In the proposed setup, a variable phase retarder is used to alternate the path of the beam.

In this paper, we also present some results of beam deflection measurements. These results provide proof that two-axial measurements are possible using only one single-axis sensor. In future, if the proposed solution is widely adopted, it could reduce the number of sensors needed in laboratories or industry, thus creating the possibility of reducing equipment costs.

## 2. Materials and Methods

### 2.1. Optical Element for the Rotation of Laser Beam Deflections

**Figure 1:**
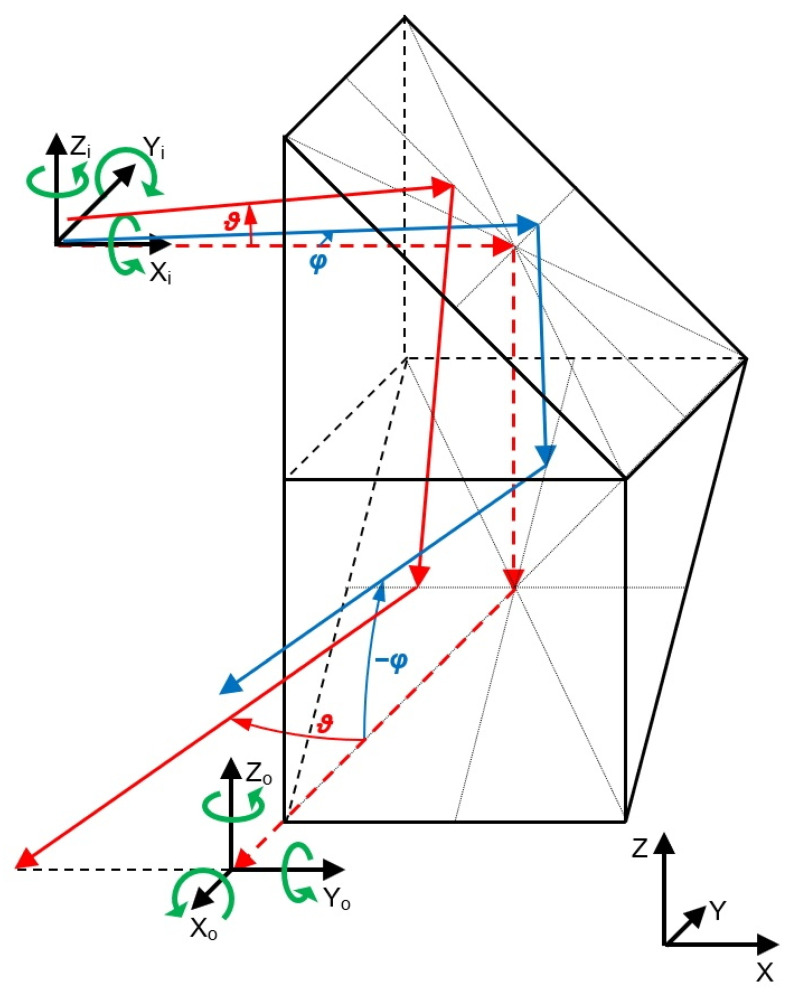
Proposed optical element that rotates the deflection of a laser beam. The green arrows show the directions of rotation with a positive sign: dashed red line—the nominal path of the beam, solid red and blue lines—the deflected beams.

The solution proposed in this paper is based on a special optical prism which rotates laser beam deflections, and this needs to be described before the solution itself is presented. This optical element consists of two right-angled prisms that are rotated by 90° in relation to each other and cemented together by an adjoining wall, as shown in Figure 1.

The dashed red line shows the nominal path of the beam, i.e., the path of the beam when it has not been deflected in any direction. If the global coordinate system XYZ is oriented as shown in the figure, the beam enters the optical element in the X-direction and leaves it in the −Y-direction. To describe the deflections of the beam, we introduce the local coordinate systems X_i_Y_i_Z_i_ and X_o_Y_o_Z_o_, where the indices i and o represent the local coordinate systems of the input and output beams, respectively. Axes X_i_ and X_o_ are positioned along the direction of the nominal path of the beam: if the nominal path of the beam is horizontal, then axes Z_i_ and Z_o_ are vertical and point upwards (parallel to the *Z*-axis of the global coordinate system).

The first case is illustrated in Figure 1 by the solid blue line. The deflection angle *φ* represents an angle of rotation around the *Z_i_*-axis. The deflection angle *φ* shown in the figure is counterclockwise, looking from above the X_i_Y_i_ plane, and we assume that it has a positive value. After passing through the optical element and undergoing two reflections inside it, the output beam is deflected in the vertical plane. In the local coordinate systems, a rotation around the *Z_i_*-axis changes to a rotation around the Y_o_-axis of the same value but with the opposite sign (clockwise looking from above the X_o_Z_o_ plane). The angle of rotation of the output beam around the Y_o_-axis has a value of −*φ* (the absolute value is the same, but the sign is negative). Hence, in the global coordinate system, a rotation around the Z-axis is changed to a rotation around the X-axis with the same absolute value but with the opposite sign.

The second case is illustrated in Figure 1 by the solid red line. The deflection angle *ϑ* is an angle of rotation around the Y_i_-axis. In this case, the deflection angle of the beam before entering the optical element has a negative value (clockwise looking from above the X_i_Z_i_ plane). The direction of the beam deflection was chosen for better visibility of the beam path, as the output beam is deflected in the horizontal plane. In the local coordinate systems, a rotation around the Y_i_-axis changes to a rotation around the Z_o_-axis with the same value and the same sign. An angle of rotation of the output beam around the Z_o_-axis has a negative value of *ϑ*. Hence, in the global coordinate system, a rotation around the Y-axis changes to a rotation around the Z-axis with the same absolute value and with the same sign.

In the above analysis, we have assumed that input beam is inclined at exactly 45° with respect to the reflecting surface.

In view of the usage of the proposed optical element, it is important that the absolute value of the rotation of the output laser beam around the Y_o_-axis (|*φ_L_*|) is equal to the absolute value of the rotation of the input laser beam around the Z_i_-axis (|*φ_E_*|). The accuracy of this conversion was determined in [26], and the error in the conversion Δ*E_C_* (the difference between the −*φ_L_* and *φ_E_* values) can be approximated by Equation (1):

Δ*E_C_* ≈ 0.018 × *R*_Y_ × *ϑ_E_*, (1)

where *R*_Y_ is the rotation of the optical element around the Y-axis relative to its nominal position in degrees, and *ϑ_E_* is the rotation of the entering laser beam around the Y_i_-axis.

There are other factors influencing the accuracy of this measurement, for example, the rotation of the optical element around the Z-axis *R*_Z_; however, these factors are less important, as their influence is smaller.

Since any significant rotation of the optical element around the Y-axis can be a major source of error in the measurement of laser beam deflection, it was decided that in the experimental setup, the optical element should have an angular adjustment in the XZ plane.

### 2.2. Method of Two-Axial Measurement with One Single-Axis Sensor

**Figure 2:**
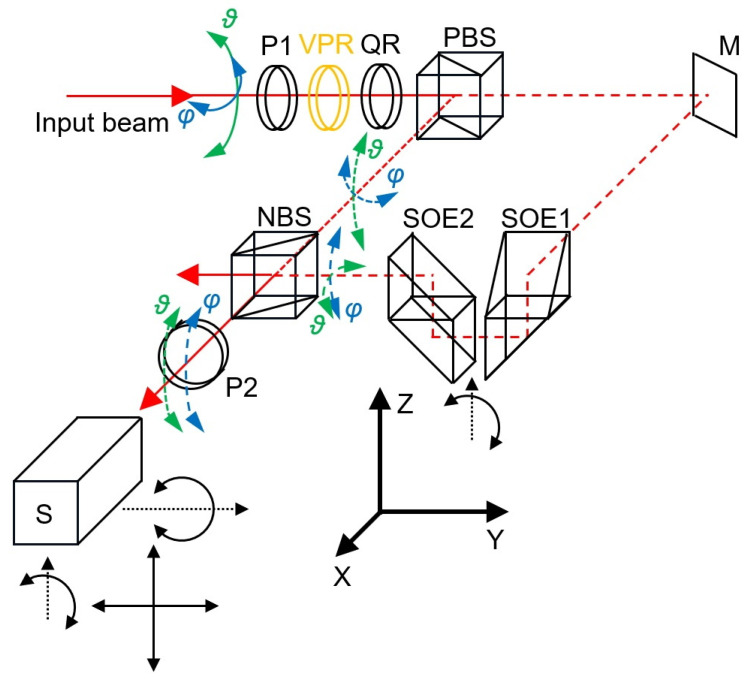
Setup for two-axial measurement using one mono-axial sensor: P1 and P2—polarisers, VPR—variable phase retarder, QR—quarter-wave retarder plate, PBS—polarising beamsplitter, NBS—non-polarising beamsplitter, M—mirror, SOE1—optical element for rotation of beam deflection, SOE2—optical element for shifting the beam, S—angular sensor. Black arrows show the directions of possible adjustments.

A schematic diagram of the proposed method of two-axial measurement with one single-axis sensor is shown in Figure 2.

The laser beam for which the deflections are measured is depicted as a solid red line. Its deflection in the vertical plane is designated as *ϑ*, while its deflection in the horizontal plane is designated as *φ*. Depending on the controlling signal sent to the variable phase retarder VPR, the beam that passes through polariser P1, the variable phase retarder VPR and the quarter-wave plate QR has a vertical or a horizontal polarisation (a more detailed description of the subsystem for altering the beam polarisation will be given in Section 2.3). If the polarisation is vertical, the beam is fully reflected by the polarising beamsplitter PBS. The path of the reflected beam is shown by the short-dashed red line. In this case, the beam enters the non-polarising beamsplitter NBS, and the transmitted part of the beam passes through the polariser P2, where it finally enters the angular sensor S. The sensor measures the vertical component (*ϑ*) of the initial beam deflection. Polariser P2 is necessary because the use of sensor S requires that the beam is polarised at an angle of 45°. A more detailed description of the sensor is given in Section 2.4.

If the polarisation of the beam entering the polarising beamsplitter PBS is horizontal, the beam is fully transmitted. Its path in Figure 2 is shown by the long-dashed red line. It is reflected by the mirror M and enters optical element SOE1, which rotates the deflection of the beam through 90°. After passing through this element, the vertical component of the beam deflection *ϑ* is replaced by the horizontal component of the initial beam deflection *φ*. After this rotation, the beam is raised by optical element SOE2 and enters the non-polarising beamsplitter BS. The reflected part of the beam passes through polariser P2 and enters sensor S. Since the vertical deflection of the beam is now equal to the horizontal deflection of the initial beam (*φ*), the sensor measuring only in the vertical plane now measures the horizontal deflection of the initial beam.

Although the optical element SOE1 changes the sign of the horizontal component of the initial beam deflection, the mirror M also changes this sign, meaning that the overall sign is not changed.

### 2.3. Subsystem for Altering the Beam Polarisation

**Figure 3:**
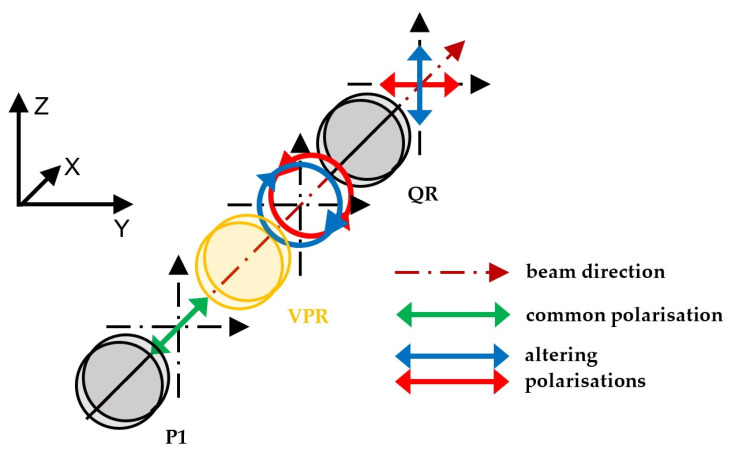
Principle of operation for the subsystem altering the beam polarisation: P1—polariser, VPR—variable phase retarder, QR—quarter-wave retarder plate.

The principle of operation for the subsystem altering the beam polarisation is shown in Figure 3.

The beam passes through polariser P1, which ensures that the beam entering the variable phase retarder VPR has a linear polarisation at an angle of 45° (shown by the green arrows in Figure 3). The variable phase retarder is applied to change the polarisation of the beam to right-hand circular (shown in the figure as a blue circle with arrows) or left-hand circular (shown in the figure as a red circle with arrows). The direction of circular polarisation depends on the amplitude of the signal controlling the variable phase retarder. The circularly polarised beam then passes through the quarter-wave plate retarder QR, which is fixed at an angle of 45°. This plate changes the right-hand circular polarisation to vertical polarisation (shown by blue arrows in the figure) and changes the left-hand circular polarisation into horizontal polarisation (shown by red arrows in the figure).

For the experimental setup described below in Section 2.5, exact values of the amplitude of the signal controlling the variable phase retarder were set in such a way as to obtain maximum light intensity, alternately, in the first or in the second arm of the setup (shown in Figure 2).

### 2.4. Single-Axis Sensor for Laser Beam Deflection

**Figure 4:**
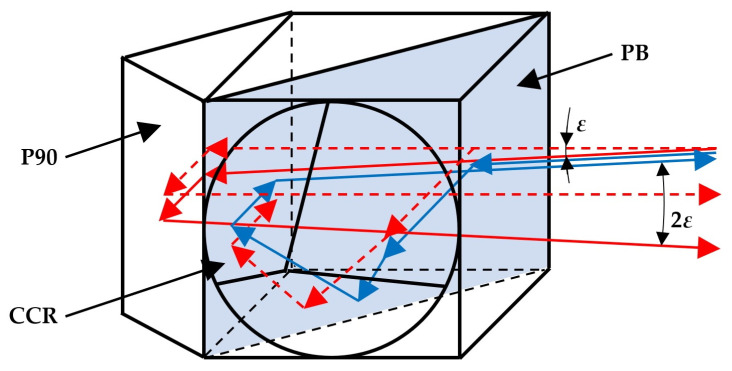
Main principle of operation of the sensor: PB—polarising beamsplitter, CCR—corner cube reflector, P90—right-angle prism.

The method proposed in this paper can be applied with (almost) any mono-axial sensor for the angular deflection (or microdeflection) of a laser beam. However, it was developed for use with the sensor described in [20,23], and the results presented here were obtained using a sensor of this type. This was an interference-based sensor, and its main principle of operation is shown in Figure 4.

The main subsystem of the applied sensor is an optical system consisting of a polarising beamsplitter PB, corner cube reflector CCR and right-angle prism P90. The beam entering this optical system must have a linear polarisation at an angle of 45°. Half of the beam passes through the polarising beamsplitter, while the other half is reflected to the corner cube reflector. After undergoing reflection in the prism and the corner cube reflector, both halves of the beam are returned to the beamsplitter. The half of the beam from the prism passes through the beamsplitter, while the half of the beam from the corner cube reflector is reflected. As a result, if the initial beam is propagating in the horizontal direction, both parts of the input beam leave the optical system in the same direction. This case is shown in Figure 4 by the dashed red line. However, if the input beam is deflected in the vertical direction (for example, downwards) by an angle *ε*, the half of the beam reflected by the prism (shown as a continuous red line) leaves the optical system still propagating downwards, while the half of the beam reflected by the corner cube reflector (shown as a continuous blue line) leaves the system parallel to the initial beam, i.e., upwards. In this case, the angle between the two beams leaving the optical system is 2*ε*. A similar situation arises if the initial beam is deflected upwards. It is important to note that the projections of the beams leaving the optical system onto the horizontal plane are always parallel (they overlap).

If two coherent beams with plane wavefronts, the same polarisation and a deflection by a small angle 2*ε* overlap, they interfere, and the interference pattern consists of fringes with a constant (period) *δ* given by Equation (2) [20]:

(2) $$\delta =\frac{\lambda }{2sin\left(\varepsilon \right)}\approx \frac{\lambda }{2\varepsilon }\;,$$

where *λ* is the wavelength of the beams. For the case of the sensor described above, the beams leaving the optical system in Figure 4 have perpendicular polarisations. To enable interference between them, an additional polariser set to 45° is used (which is not shown in the figure). A photodetector system is placed behind this polariser. This system enables the fringe constant *δ* to be determined, which then allows for the angle *ε* to be calculated.

Various photodetector systems can be used; for example, a CCD camera can be applied. In the sensor used to verify the proposed method of two-axial measurements, the same photodetector system (eight photodiodes in a row) was applied with the main settings reported in [23]. Each photodiode was 200 µm wide. If the value of the signal from the first photodiode is labelled as *I*_1_, the value of the signal from the second photodiode as *I*_2_, and so on, then the function *F* related to the fringe constant is given by Equation (3) [20]:

(3) $$F=\frac{\left(I_{7}-{\;I}_{5}-I_{4}+I_{2}\right)}{1.85\left(I_{4}+I_{5}\right)-I_{3}-I_{6}}.$$

The values of this function are normalised to the range [−1, 1]. The value of the angle *α* can then be calculated according to Equation (4) (the function *F* after normalisation is labelled as *F_n_*) [20]:

(4) $$\propto =25.1287F_{n}^{7}+0.648825F_{n}^{6}-18.1042F_{n}^{5}-0.951463F_{n}^{4}+16.6809F_{n}^{3}+0.206846F_{n}^{2}+63.9919F_{n}.$$

The value of the angle *α* changes by the same amount as the angle *ε*, but also contains an initial angle *ε*_0_ that is related to the centre of the measurement range. In this configuration, the sensor does not enable absolute measurements—these require the configuration with the CCD camera.

Figure 2 shows the four adjustment axes of the sensor position and orientation. Linear adjustments should be made so that the spot of the laser beam is located at the centre of the photodetector system. The adjustment around the Z-axis should be done so that the optical path lengths for the beams reflected by the prism and the corner cube reflector are equal. The adjustment around the Y-axis should be made so that the measured angle values are within the measurement range (that is equal approximately 350 µrad). The precision of these adjustments influences the accuracy of the sensor.

### 2.5. Setup Used for Experimental Verification of the Proposed Method

**Figure 5:**
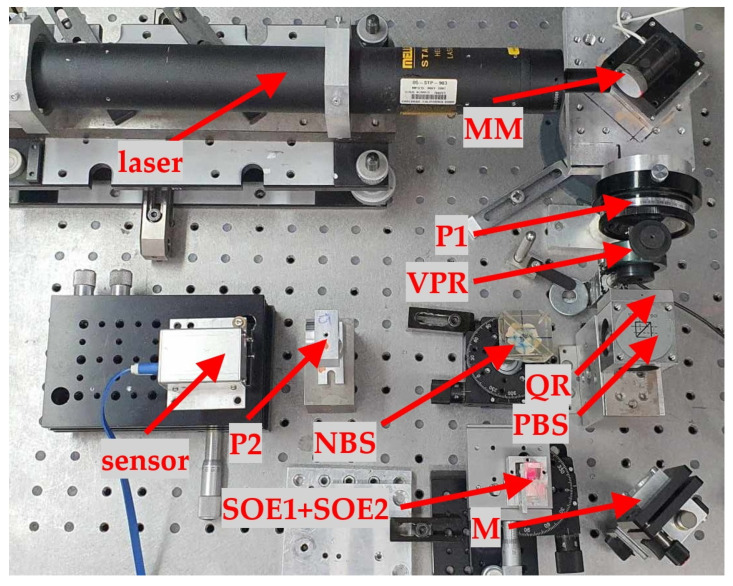
Setup used for experimental verification of the proposed method: P1 and P2—polarisers, VPR—variable phase retarder, QR—quarter-wave retarder plate, PBS—polarising beamsplitter, NBS—non-polarising beamsplitter, M—fixed mirror, SOE1—optical element for rotation of beam deflection, SOE2—optical element for raising the beam, MM—mirror on piezotranslator controlling the beam.

To verify the effectiveness of the proposed method, the setup presented in Figure 5 was assembled. The labels are consistent with those of Figure 2, and the setup included polarisers P1 and P2, variable phase retarder VPR, quarter-wave plate QR, polarising beamsplitter PBS, non-polarising beamsplitter PS, fixed mirror M, optical elements SOE1 and SOE2, and the sensor. This part of the larger setup was assembled as shown in Figure 2. For the variable phase retarder, an ARCoptix Variable Phase Retarder was used.

The source of the light was a He–Ne laser containing a beam expander and collimator. The collimator was required because the interfering beams in the sensor need to have approximately plane wavefronts. The laser was the most important source of error during the experimental verification of the proposed method. Usually, the angular component of pointing stability specified for good quality lasers is at the level of few dozen µrad in one hour. In practice, short-term pointing stability of a He–Ne laser is usually much better, but it still has to be taken into account. We decided to include the errors caused by this factor into measurement of random errors. Based on our earlier experiences with He–Ne lasers of a comparable quality [23], we decided to accept the errors caused by laser pointing instability at a level not exceeding 2 µrad.

To deflect the laser beam through the set angles (*ϑ* in the vertical plane and *φ* in the horizontal plane), a mirror on a two-axial, rotational piezotranslator (MCL Nano-MTA2 [27] with MCL Nano-drive 2 [28]) was used. In the horizontal plane, a factor equal to the square root of two was applied, as the controlling mirror was rotated through an angle of 45°. According to the manufacturer’s specification, resolution of the applied piezotranslator is equal to 4 nrad, while maximum positioning error is equal to 1 µrad in the full range of 2 mrad. Its sensitivities to voltage signals had been provided by the manufacturer, but additional verification was also performed using an autocollimator. It had to be considered that the amplification in the vertical and horizontal planes varied [26], while the voltage values (before applying the factor of the square root of two) were set the same for both axes. For this reason, the set values of *ϑ* and *φ* were different: *ϑ* nominally ranged from 0 to 17.95 µrad, while *φ* nominally ranged from 0 to 15.11 µrad. These small ranges for the set values in the experiments were selected since measurements of similar angles were performed in [23]. The measurement errors caused by the piezotranslator were included in overall random errors. However, because only a tiny part of the piezotranslator range was used, it is probable that these errors did not exceed 0.2 µrad, even taking into account the error multiplication caused by the beam reflection.

A total of four experiments were performed. In Experiment 1, only the value of *ϑ* was measured. The variable phase retarder was set so that the beam was reflected by the polarising beamsplitter directly to the sensor, and there was no change in the amplitude of the signal controlling the variable phase retarder. This experiment was carried out to test the performances of the sensor and the laser themselves. Although earlier tests of this type of sensor had been done, this experiment was necessary because the accuracy of the sensor depends on the precision of its adjustment.

In Experiment 2, both angles were measured. They were changed simultaneously from 0 to 17.95 µrad for *ϑ* and from 0 to 15.11 µrad for *φ*, which was performed incrementally with constant steps (where the step was constant for a given direction, but different for each direction). After each step, a measurement was made in both directions. There were 19 steps and 20 measurements for each angle (as the first measurements were made for angles of 0). For this experiment, two runs were performed.

In Experiment 3, both angles were measured, but only *φ* was changed, whereas in Experiment 4 both angles were measured, while only *ϑ* was changed. The main purpose of conducting these two experiments was to check whether results of the angle *φ* measurement in Experiment 2 (i.e., for increasing *ϑ*) would differ from results of the *φ* measurement obtained in Experiment 3 (i.e., for constant or quasi-constant *ϑ* angle).

If the measured value of *φ* angle depended on the value of *ϑ* angle, the *φ* values obtained in Experiment 3 would differ from these obtained in Experiment 2. But this effect was not observed. In particular, for the largest measurement results in Experiment 3, *φ* values were obtained in a range not exceeding the corresponding values from the two runs in Experiment 2.

Experiment 4 was similar, but we had to prove that the φ did not affect the measurement of *ϑ*.

## 3. Results

In the following section, the results of performed experiments are presented. The solid lines show obtained results, while thin, dotted lines in figures show ± extended uncertainty (*k* = 2) zones. The colours of these lines indicate the measured angles or experiment to which they apply. The calculations of the uncertainty values, which were different for two of the measured angles, are presented in Section 3.2, following the section containing the obtained results.

### 3.1. Results Overwiev

**Figure 6:**
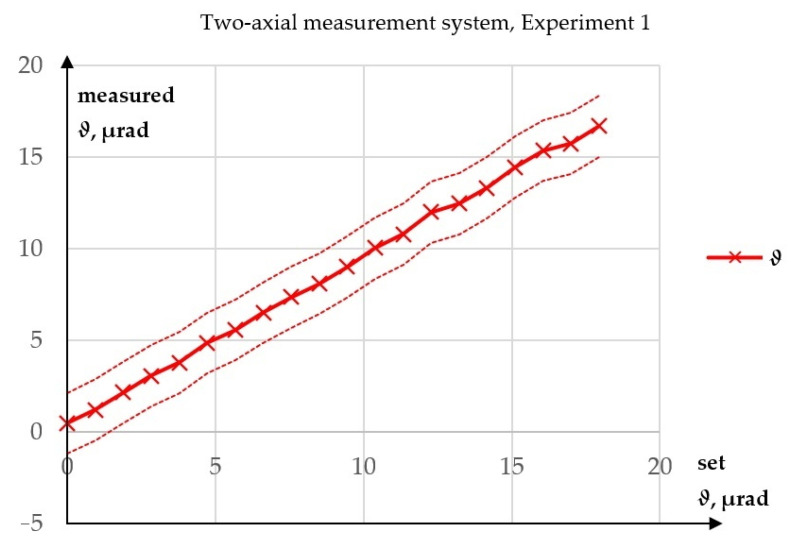
Results of Experiment 1.

The results of Experiment 1 are shown in Figure 6. In this experiment, the maximum absolute error—the greatest absolute value of the difference between a measured value and a set value—in the measurement of *ϑ* was 1.24 µrad. It may be more significant that the R^2^ value for a linear fit (calculated using Statgraphics Centurion 19 v19.1.3 software) was 99.9274%. The linear characteristic of a measurement system is important, because even if there were some systematic errors, for example, caused by an error in the determination of the piezotranslator amplification, the proportional response of the measurement system verifies the performance of the method.

**Figure 7:**
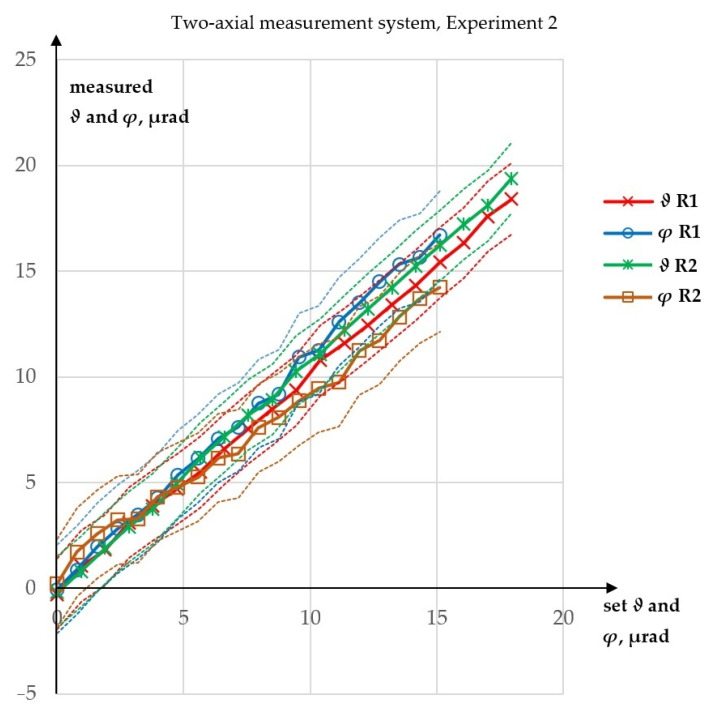
Results of Experiment 2: R—run.

The results of Experiment 2 are presented in Figure 7.

In the first run of Experiment 2, the maximum absolute error in the measurement of *ϑ* was 0.59 µrad, while the absolute error in the *ϑ* measurement was 1.81 µrad. The R^2^ values for the linear fits were 99.9277% for *ϑ* and 99.7965% for *φ*. In second run of Experiment 2, the maximum absolute error in the measurement of *ϑ* was 1.45 µrad, while that for *φ* was 1.39 µrad. The R^2^ values for the linear fits were 99.9316% for *ϑ* and 99.7383% for *φ*. None of the errors in Experiment 2 exceeded 2 µrad.

**Figure 8:**
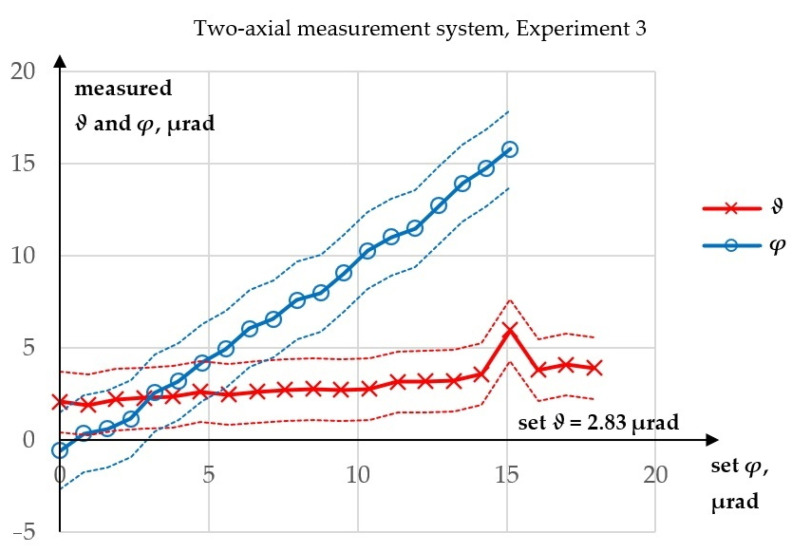
Results of Experiment 3.

**Figure 9:**
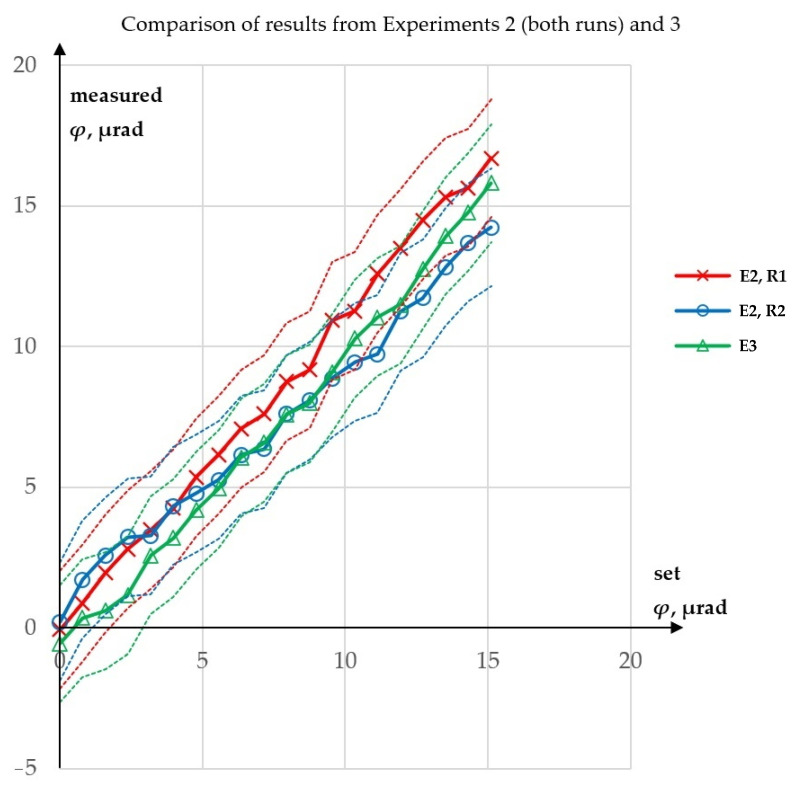
Comparison of results from Experiments 2 and 3: E—experiment, R—run.

Experiment 3 was performed to test whether the value of *ϑ* influenced the measurement of *φ*. It was expected that the measurement of *φ* for constant *ϑ* might give different results than for increasing *ϑ*. The results of this experiment are presented in Figure 8, while a comparison of the *φ* values obtained from Experiments 2 and 3 is shown in Figure 9.

In Experiment 3, the maximum absolute error in the measurement of *φ* was 1.22 µrad, while the R^2^ value for a linear fit was 99.6856%. The maximum absolute error in the measurement of *ϑ* was 3.15 µrad. However, one measurement of *ϑ* was clearly an outlier. It was proved by statistical methods that the 17th observation in the data shown in Figure 8 by the red line was an evident outlier caused by unidentified random disruption and this data point was removed from the data set. After elimination of this data point, the maximum absolute error in *ϑ* decreased to 1.27 µrad. A drift of the measured *ϑ* values was observed, but the differences were within the range of the expected laser pointing angular drift. Additionally, the next experiment showed that there was no influence of *φ* on the measurement of *ϑ*. There is also no reason *φ* would affect the measurement of *ϑ*, because while *ϑ* is being measured, the beam is only reflected by the polarizing beamsplitter. The time between two data points in a single experiment was approximately 1 min. Taking into account all of the above, it was concluded that there was no real cross talk. The increase in *ϑ*, visible in Figure 8, was caused by the angular component of laser pointing instability. Alternatively, there could have been a change in the direction of the laser beam caused by the thermal drift in the setup. But in both cases, there was drift of the laser beam direction.

The maximum difference between corresponding *φ* values obtained from Experiments 2 and 3 was 2.85 µrad. However, the graph in Figure 9 shows that the source of this relatively large maximum difference lay, not in the influence of *ϑ*, but in the repeatability of the measurement itself. When the corresponding *φ* values obtained from both runs of Experiment 2 were averaged, the maximum difference relative to the results from Experiment 3 decreased to 1.84 µrad. Moreover, in neither experiment did the maximum absolute value of error of the measurement of *φ* (i.e., maximum difference between measured *φ* and set *φ*) exceed 1.81 µrad.

If the measured value of *φ* angle depended on the value of *ϑ* angle, the *φ* values obtained in Experiment 3 would differ from these obtained in Experiment 2. But this effect was not observed. In particular, for the largest measurement results in Experiment 3, *φ* values were obtained in a range not exceeding the corresponding values from the two runs in Experiment 2. Eventually, because the measurement of *φ* gave similar results for quasi-constant *ϑ* and for increasing *ϑ*, it was concluded that *ϑ* had no influence on the measurement of *φ*.

**Figure 10:**
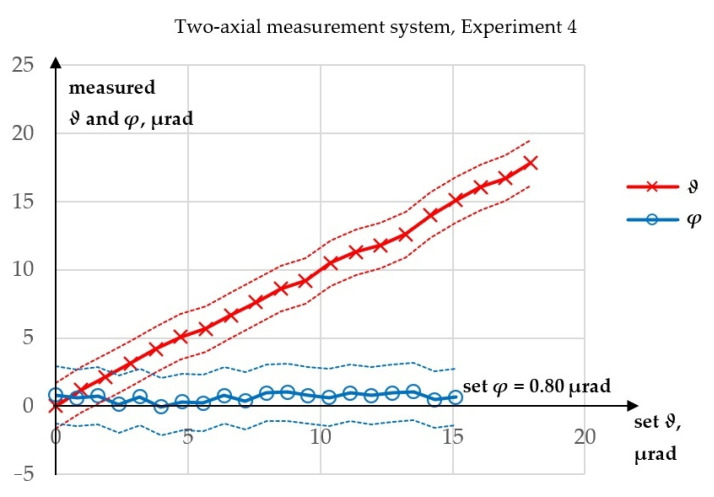
Results of Experiment 4.

**Figure 11:**
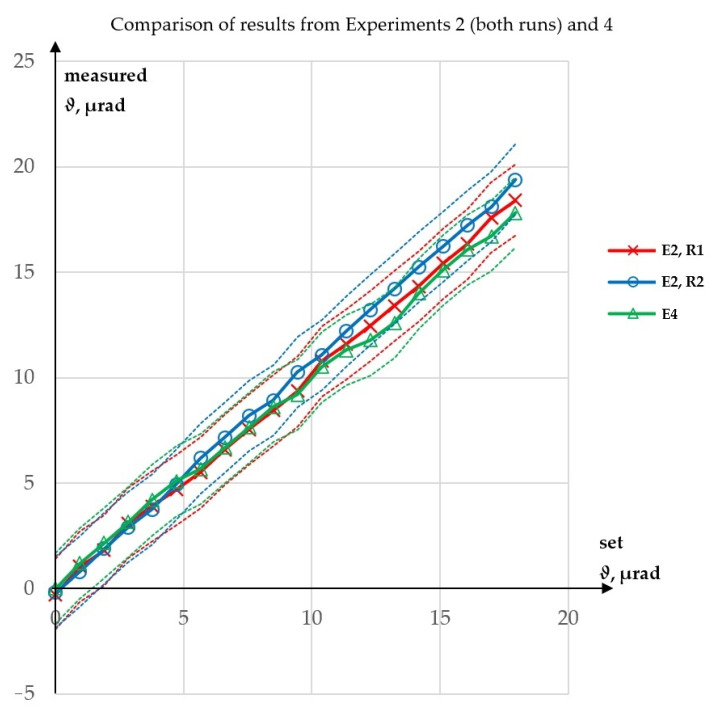
Comparison of results from Experiments 2 and 4: E—experiment, R—run.

Experiment 4 was performed to test whether the value of *φ* influenced the measurement of *ϑ*. Contrary to the case of the influence of *ϑ* on the measurement of *φ*, there was no theoretical basis for expecting an influence of *φ* on the measurement of *ϑ*. The results of this experiment are presented in Figure 10, while a comparison of the *ϑ* values obtained from Experiments 2 and 4 is shown in Figure 11.

In Experiment 4, the maximum absolute error in the measurement of *ϑ* was 0.62 µrad, while the R^2^ value for a linear fit was 99.8529%. The maximum absolute error in the measurement of *φ* was 0.84 µrad. The maximum difference between the corresponding values of *ϑ* obtained from Experiments 2 (both runs) and 4 was 1.62 µrad, a value similar to those obtained in the single experiments. Therefore, it was concluded that *φ* had no influence on the measurement of *ϑ*: the measurements of *ϑ* gave similar results for constant *φ* and for increasing *φ*. This was expected, because the theoretical model allows only the influence of *ϑ* on the measurement of *φ*. In Figure 10, no angular drift of the laser beam is visible in the measurements of *φ* angle. This does not mean that there was no angular drift of the laser beam. Since the spatial direction of the laser beam is random the beam deflection might have been present at that time in the vertical plane.

Since, after elimination of the outlier, the error in the measurement did not exceed ±2 µrad—the expected laser pointing instability—in either direction (vertical or horizontal), these results prove that the proposed method is effective. In order to estimate the uncertainty of the above measurement results, the simplified analysis is presented below.

### 3.2. Uncertainty Analysis

**Figure 12:**
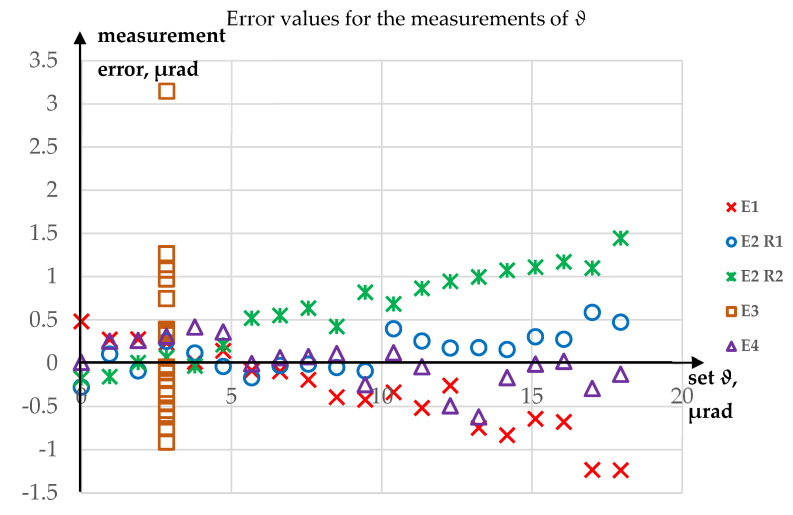
Error value for the measurements of *ϑ*.

**Figure 13:**
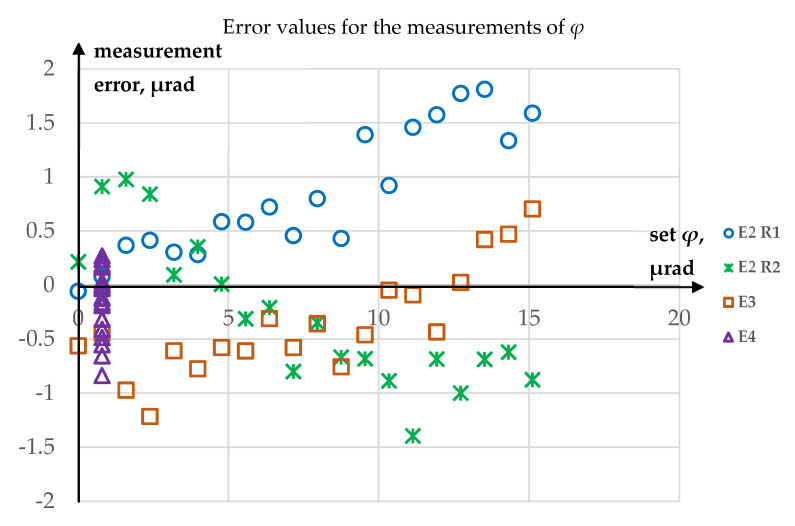
Error value for the measurements of *φ*.

Since the carried-out research was only a feasibility study, there are no results of multiple repetitions of each of the experiments. In order to assess uncertainty of measurements, maximum absolute values of errors of *ϑ* and *φ* angle measurements were determined. As it had been proved that the outlier in the results of the measurement of *ϑ* in Experiment 3 could be removed, this has not been included in the uncertainty calculations. For *ϑ* angle, the maximum absolute value of measurement error Δ*ϑ* = 1.45 µrad, while for *φ* angle, the maximum absolute value of measurement error Δ*φ* = 1.81 µrad. Plots of the obtained errors for *ϑ* and *φ* angles are shown, respectively, in Figure 12 and Figure 13.

Assuming that errors of measurement have rectangular distribution, the standard uncertainty of *ϑ* measurement *u*(*ϑ*) is equal:

(5) $$u\left(\vartheta \right)=\frac{∆\vartheta }{\sqrt{3}}\;$$

while the extended uncertainty of *ϑ* measurement *U*(*ϑ*) is:

*U*(*ϑ*) = *u*(*ϑ*) × *k*, (6)

where *k* is coverage factor. For *k* = 2 *U*(*ϑ*) = 1.67 µrad.

Extended uncertainty of *φ* angle measurement *U*(*φ*) was calculated in the same way and was equal 2.09 µrad.

**Figure 14:**
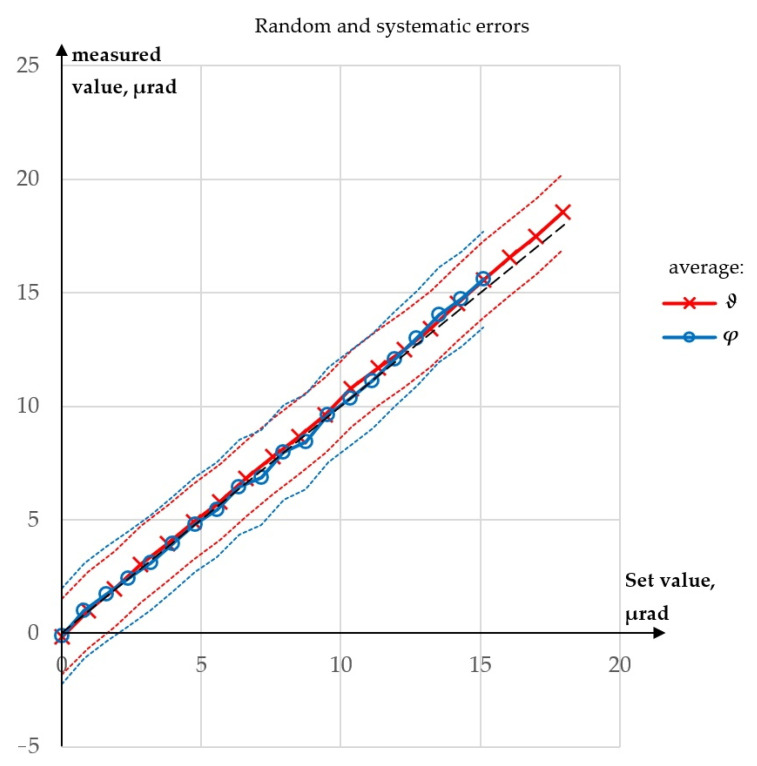
Average values of the results from 3 runs with extended uncertainties: black, dashed line—*y* = *x* line.

In Figure 14, average values of the measured *φ* for Experiments 2 and 3, and average values of the measured *ϑ* for Experiments 2 and 4, are shown with corresponding extended uncertainty zones.

As can be seen, the random errors were much higher than the systematic errors. After averaging only three sets of results, maximum absolute error in the measurement of angle *ϑ* dropped to 0.60 µrad, while maximum absolute error in the measurement of *φ* angle dropped to 0.51 µrad. Unfortunately, in this case, performing multiple measurements would not improve the accuracy, as the most important source of error was laser pointing instability which generates a higher error the longer observation time. Over a short period of time this source of error has random character, but over a longer period of time, it will generate systematic error.

## 4. Discussion

The proposed method of two-axial measurement of a laser beam angular (micro) deflection was successfully verified experimentally. The use of only one single-axis sensor rather than two such sensors could decrease the cost of measurement equipment and make the required adjustments easier and quicker.

The range of set values in the experiments was selected to allow the proposed method to be used for testing the rotational errors of coordinate measuring machines [23]. The obtained measurement errors provide evidence that this type of application is possible. The accuracy of a two-axial system with only a single sensor appears to be not worse than the accuracy of the two-sensor system presented in [23]. Although, in the case of application, in testing, of rotational errors of coordinate measuring machines, metrological properties of the setup presented in the paper are not yet satisfactory, the obtained uncertainty of angle measurement is smaller than possible changes of rotational errors of a machine caused by fixing an additional mass (for example, a piece of a multi-axis calibrator) to the machine ram [23]. The proposed method can be applied using other sensors of the angular deflection of a laser beam and in other fields of science or engineering. It is possible that in the case of higher measurement ranges, the influence of the deflection in the perpendicular direction on the measurement of deflection will become important; if so, the simple formula in Equation (1) can be used for a numerical correction. However, this problem should not occur for small measurement ranges. For example, if the crucial optical SOE1 (Figure 2) is rotated by 1° around the vertical axis, vertical deflection of a laser beam by 100 µrad causes an error equal to only 1.8 µrad in the measurements of horizontal deflection [26]. In the case of larger beam deflection the error can be reduced using Equation (1). In future setups it should be possible to adjust the optical element for rotation of beam deflection with a precision much better than the assumed 1°. In conclusion, it is possible to measure laser beam angular deflections in two perpendicular directions by applying only one mono-axial sensor. The solution involves alternately directing the laser beam to the sensor through two arms of the measurement system, where only one of them contains an optical element to rotate the deflection.

## 5. Patents

The method described in this paper is patented in Poland. The patent number is PL 241303 and the patent title is ‘Method for measuring angular deviations of a laser beam and the optical system for measuring angular micro-deviations of a laser beam’.

## Data Availability

Publicly available datasets were analyzed in this study. This data can be found here: https://wutwaw-my.sharepoint.com/:x:/g/personal/marek_dobosz_pw_edu_pl/EUAjVK9RU3FIh9tjSqDzojsBZvX7vGr2RzUI7hqo7-NxAg?e=E1JGEs (accessed on 16 November 2023).
